# Evaluation of Corneal Higher-Order Aberrations by Scheimpflug–Placido Topography in Patients with Different Refractive Errors: A Retrospective Observational Study

**DOI:** 10.1155/2019/5640356

**Published:** 2019-06-02

**Authors:** Mohamed Anbar, Engy Mohamed Mostafa, Ashraf Mostafa Elhawary, Islam Awny, Mahmoud Mohamed Farouk, Amr Mounir

**Affiliations:** ^1^Assistant Professor, Sohag Faculty of Medicine Ophthalmology Department, Sohag, Egypt; ^2^Lecturer, Sohag Faculty of Medicine Ophthalmology Department, Sohag, Egypt

## Abstract

**Purpose:**

To report the characteristics of anterior and posterior corneal high-order aberrations in patients with different refractive errors.

**Setting:**

This study was conducted at Sohag Refractive Center, Sohag, Egypt.

**Design:**

This is a retrospective observational study.

**Methods:**

This study evaluated 750 patients (750 eyes) who were seeking refractive surgery. The eyes were stratified into five groups (150 eyes/group) based on refractive error: mild-to-moderate myopia, high myopia, hyperopia, simple myopic astigmatism, and simple hypermetropic astigmatism. All patients were subjected to comprehensive ophthalmological examination including corneal topography and corneal aberrometry using the Scheimpflug–Placido topography (Sirius, CSO, Italy).

**Results:**

Coma aberration was statistically significant when compared in all five groups (*P*=0.01). It was highest in the hypermetropia group (0.26 ± 0.12 *μ*m) but lower in the moderate myopia, high myopia, myopic astigmatism, and hypermetropic astigmatism groups. Spherical aberration was lowest in the hypermetropia group and significantly different from that in the other groups. Trefoil was statistically insignificant when all groups were compared (*P*=0.062) but was highest in the myopic astigmatism group (0.24 ± 0.25 *μ*m). Total RMS peaked in the hypermetropia group (0.99 ± 0.70).

**Conclusions:**

In normal corneas and regular refractive errors, the cornea-induced high-order aberration was minimal, and all types of refractive errors were associated with certain types of high-order aberrations, with a significant increase in spherical aberration in the hypermetropia group.

## 1. Introduction

The human eye is a complicated optical system with different aberrations, and these aberrations are some of the limiting factors of visual quality [1–4]. The optical quality of the eye is limited by different factors including optical aberrations, diffraction, and scatter [5]. The air cornea is the first and most important ocular refractive interface with major effect on total refraction due to the large difference in the refractive index [6].

The surge in wavefront-guided corneal refractive surgery [7, 8], aberration-correcting contact lenses [9], and wavefront-based custom intraocular lenses [10] has shed more light over high-order aberrations (HOA). HOAs form a minor part of the ocular aberrations where low-order aberrations (myopia (positive defocus), hyperopia (negative defocus), and regular astigmatism) constitute more than 90% of all wave aberrations [11, 12]. HOA impairs retinal image quality in the form of glare and halos, yet they cannot be corrected with sphere and cylinder lenses [13]. Attention to HOAs after laser corneal refractive surgery has recently become one of the crucial issues when assessing the quality of laser refractive methods [14, 15].

Corneal topography devices traditionally provide corneal aberrometry using special algorithms based on elevation data; however, different ocular aberrations have recently been determined using data from aberrometers [16].

The Sirius Scheimpflug–Placido topographer (Costruzione Strumenti Oftalmici) combines a rotating Scheimpflug camera and Placido-disk technology. In a single scan, it provides anterior segment imaging and measurements, anterior and posterior corneal topography, wavefront analysis, and corneal pachymetry [17].

Several studies have investigated the relationship between HOAs and different refractive errors; however, their results were conflicting [18–20]. Knowledge about the distribution of HOAs associated with refractive errors may help to produce more accurate and optimum corrections when using new techniques in refractive surgeries. Also, widening the knowledge of HOA patterns may help in the early diagnosis of keratoconus [21].

The aim of this study was to evaluate corneal HOAs in patients with different refractive errors.

## 2. Patients and Methods

This is a retrospective observational comparative study of 750 candidates who seeked refractive surgery at Sohag Refractive Center, Sohag, Egypt, between January and May 2018. One eye from each candidate was used (750 eyes) according to a random-number sequence. Eyes were divided into five groups according to their refractive errors: mild-to-moderate myopes (−1 : −5.9D) (202 eyes), high myopia (−6 : −9D) (166 eyes), hyperopes (+1 : +4D) (101eyes), simple myopic astigmatism (cylinder ≥ -1D) (199 eyes), and simple hypermetropic astigmatism (cylinder ≥ +1D) (82 eyes). Exclusion criteria were previous ocular surgery, glaucoma patients, systemic diseases such as diabetes, keratoconus or keratoconus suspect, amblyopic eyes, and patients with BCVA not achieving 6/6. Candidates wearing contact lenses were instructed to discontinue the use of soft contact lenses for 2 weeks before examination and those wearing rigid gas-permeable contact lenses, for 4 weeks. All patients were subjected to routine comprehensive preoperative examinations. Corneal topography and corneal aberrometry were performed on all patients using Sirius's Scheimpflug–Placido topography (CSO, Florence, Italy). The CSO topography system analyzed a total of 6144 corneal points of a corneal area within a circular annulus outlined by an inner radius of 0.33 mm and an outer radius of 10 mm with respect to the corneal vertex.

The patient's eye was aligned through the visual axis using a central fixation light. Patients were informed to blink between shots to keep the tear film intact. The eye movement of the subject was regularly tracked by the system, and quality factor was automatically evaluated. All scans were centered on the center of the pupil. Mesopic pupil diameter was acquired in a dark room with the disc illuminated in a manner to bring ambient light intensity to 4.0 lux as advised by the manufacturer [22].

Anterior and posterior corneal aberrometry data were collected from the Sirius over a 5 mm diameter. Root mean square (RMS) total HOAs, RMS coma, RMS trefoil, RMS astigmatism, and RMS spherical aberrations (SA) were evaluated.

The Ethical Committee of Sohag Faculty of Medicine approved this study, and the tenets of the Declaration of Helsinki were followed.

## 3. Statistical Analysis

Data were analyzed using STATA intercooled version 12.1. Quantitative data were represented as either mean and standard deviation or median and range. Student's *t*-test and ANOVA test were used to compare variables of five and two groups, respectively. In cases when the data were not normally distributed, the Kruskal–Wallis and Mann–Whitney tests were used to compare five and two groups, respectively. *P* value < 0.05 was considered significant.

## 4. Results

A total of 750 eyes from 750 patients (393 males (52.4%) and 357 females (47.6%)) were enrolled in this study. The age of the subjects ranged from 18 to 41 years, with a mean age of 27.8 ± 9.4 years. The mean age of the hypermetropia group was slightly higher (30.79 ± 11.56 years); however, this difference was not statistically significant (*P*=0.43).  Table 1 shows demographic and refractive data of the five groups.

Mean and standard deviation of HOA of anterior and posterior corneal aberrations of all groups are summarized in Table 2.

**Figure 1:**
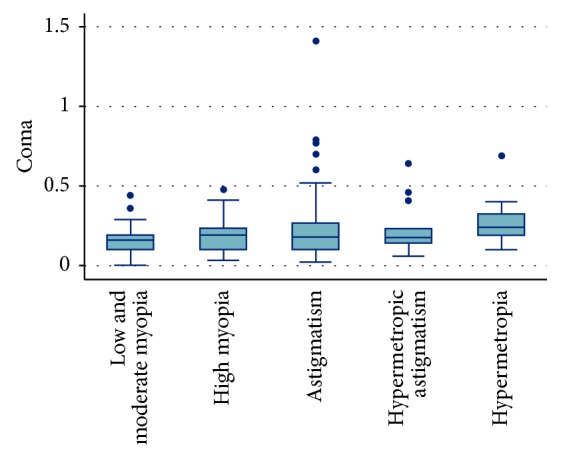
Coma distribution among different groups.

Statistical analyses of anterior corneal HOAs in the different groups of refractive errors revealed that spherical aberration was at its lowest positive level in the hypermetropia group (0.04 ± 0.02 *μ*m), increasing in hypermetropic astigmatism, myopic astigmatism, high myopia, and low and moderate myopia groups (0.09 ± 0.05, 0.14 ± 0.06, 0.16 ± 0.06, and 0.17 ± 0.05 *μ*m, respectively). The difference was statistically significant when comparing all groups together (*P*=0.0001) and when comparing the hypermetropia group with the rest of the groups. Coma aberration was statistically significant when compared in all five groups (*P*=0.01). It was highest in the hypermetropia group (0.26 ± 0.12 *μ*m) but lower in the low and moderate myopia, high myopia, myopic astigmatism, and hypermetropic astigmatism groups (0.16 ± 0.09, 0.19 ± 0.11, 0.23 ± 0.22, and 0.23 ± 0.16 *μ*m, respectively). In addition, when comparing two groups individually, coma aberration was statistically significant between the low and moderate myopia and hypermetropia groups (*P*=0.0002) (Figure 1).

**Figure 2:**
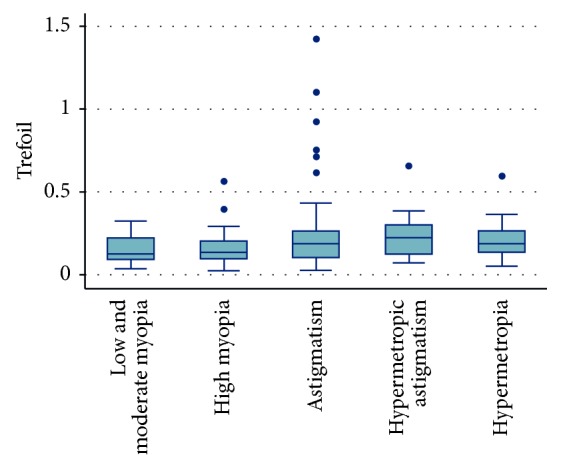
Trefoil distribution among different groups.

Trefoil was not statistically significant when all groups were compared (*P*=0.062). It was highest in the myopic astigmatism group (0.24 ± 0.25 *μ*m) and lower in the low and moderate myopia, high myopia, hypermetropic astigmatism, and hypermetropia groups (0.15 ± 0.08, 0.16 ± 0.11, 0.23 ± 0.14, and 0.21 ± 0.12 *μ*m, respectively) (Figure 2).

**Figure 3:**
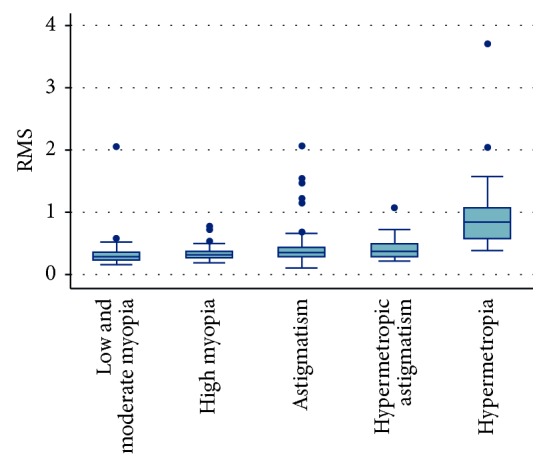
RMS distribution among different groups.

Total RMS peaked in the hypermetropia group (0.99 ± 0.70) and was lower in the low and moderate myopia, high myopia, myopic astigmatism, and hypermetropic astigmatism groups (0.34 ± 0.13, 0.34 ± 0.33, 0.44 ± 0.34, and 0.41 ± 0.22, respectively). Total RMS in the hypermetropia group was statistically significant when compared with each of the other groups individually (*P*=0.0001) (Figure 3).

As regards the posterior corneal HOA, none of the variables were significantly different in any of the refractive groups.

## 5. Discussion

This study assessed the cornea as one of the participating factors in the induction of HOAs in patients with various refractive errors. Most studies investigated ocular HOA, but we aimed at knowing the contribution of the cornea. The anterior corneal surface is the most crucial refractive interface of the eye due to the large difference in refractive index between air and cornea [1, 23]. The anterior corneal surface is almost 14 times more powerful than the posterior surface as the ratio of refractive indices between air and the anterior corneal surface is 1.0/1.376 and between the aqueous and the posterior corneal surface is 1.376/1.336 [6]. This explains the much lower levels of HOA at the posterior corneal surface and its insignificance between refractive groups.

The results showed that spherical aberration was significantly lower in the hypermetropia group, both when comparing all the groups together and when comparing the hypermetropia group with the other groups individually. Coma aberration was high in all groups and significant when the hypermetropia group was compared with the low and moderate myopia group. While trefoil aberration was highest in the myopic astigmatism group and lowest in the low and moderate myopia group, it was not statistically significant when comparing the groups altogether or when comparing two groups individually. Total RMS, RMS SA, and RMS coma were highest in the hypermetropia group and lowest in the low and moderate myopia group.

Some studies showed no significant correlation between HOAs and the amount or type of refractive error [18–20], while others concluded that there was a strong correlation between HOAs and myopia. Others noted that hyperopes had the highest levels of aberrations [16].

Therefore, the results were conflicting in this regard. Previous studies [24–30] reported the wavefront HOAs using different types of aberrometers. Khan et al. [25], Hashemian et al. [27], and Bisneto et al. [28] agreed that higher levels of spherical aberrations were found in hyperopes, while Yazar et al. [26] concluded that higher myopia had slight HOAs. Netto et al. [29] did not find a correlation between the degree of refractive error and HOAs.

The study by Philip et al. [31] calculated corneal HOAs from corneal topography and compared total, anterior corneal, and lenticular HOAs in emmetropic, myopic, and hypermetropic patients. Their results showed that hyperopic eyes (0.083 ± 0.05 *μ*m) had more positive total ocular primary spherical aberrations than emmetropic (0.036 ± 0.04 *μ*m) and myopic eyes; however, they also observed no significant difference with regards to anterior corneal spherical aberrations. Llorente et al. [32] found that hyperopic eyes tended to have higher (less negative) Q aberrations and higher total and corneal spherical aberrations than myopic eyes. Similar to our study, they also used corneal topography to estimate corneal aberrations.

Some other studies were only concerned with the evaluation of myopia-related HOAs. Kasahara et al. [33] estimated HOAs in patients with pathological myopia and found that highly myopic eyes had more HOAs than emmetropic eyes because of the increased internal aberrations. Karimian et al. [34] also concluded that primary horizontal trefoil, spherical aberrations, and primary vertical coma were the predominant HOAs in eyes with myopic astigmatism.

Astigmatism-induced HOAs were also investigated by some researchers. Leung et al. [35] studied myopic astigmatism versus simple myopia and found that myopic astigmatism corneas had more oblate nasal and temporal corneal shapes and showed significantly more negative Y trefoil and more positive vertical coma. The age of their studied population was relatively high (50–70 years). Conversely, Zhao et al. [36] only evaluated spherical aberrations and found that spherical aberrations of astigmatic corneas were similar to those of nonastigmatic corneas.

In this study, the mean age of the hypermetropia group (26.33 ± 12.38 years) was higher than the other groups and this might explain the increased spherical aberrations present in this group. This finding corroborates previous studies [37, 38], that assessed optical changes in the cornea with age, while other studies suggested that the ocular coma increases with age [39].

The main drawbacks of comparing results of other studies as regards this issue are the use of different measuring aberrometers, lack of equal pupil size in the measured eyes, different age groups, and different visual and refractive statuses of participants. All these factors affect reliable comparison and necessitate caution. Further research would be beneficial to study the concept of refraining of doing refractive surgery to patients with high HOA as in hypermetropes with HOA of 0/99 or more.

In conclusion, in normal corneas and ordinary refractive errors, the cornea-induced HOA is minimal and all types of refractive errors are almost identical at inducing certain types of HOAs.

## Figures and Tables

**Table 1:** Demographic and refractive data of all groups.

	Age	M/F	Sphere	Cylinder	Spherical equivalent
Low and moderate myopia	27.48 ± 9.51	107/95	−2.5 ± 2.8	−0.4 ± 0.4	−2.7 ± 1.50
High myopia	26.3 ± 9.9	97/87	−7.6 ± 1.8	−0.53 ± 0.32	−7.8 ± 1.50
Myopic astigmatism	27.1 ± 6.9	91/108	−4.2 ± 2.1	−2.1 ± 1.9	−5.25 ± 1.06
Hypermetropic astigmatism	29.2 ± 9.2	33/49	3.2 ± 2.8	1.8 ± 2.2	4.1 ± 2.1
Hypermetropia	30.79 ± 11.56	40/61	3.7 ± 2.2	0.26 ± 0.5	3.83 ± 2.31

**Table 2:** Corneal HOAs of all groups.

Group	RMS SA	RMS coma	RMS trefoil	Total RMS
*RMS of anterior corneal aberrations (mean ± SD)*				
Low and moderate myopia	0.17 ± 0.05	0.16 ± 0.09	0.15 ± 0.08	0.34 ± 0.32
High myopia	0.16 ± 0.06	0.19 ± 0.11	0.16 ± 0.11	0.34 ± 0.13
Myopic astigmatism	0.14 ± 0.06	0.23 ± 0.22	0.24 ± 0.25	0.44 ± 0.34
Hypermetropic astigmatism	0.09 ± 0.05	0.23 ± 0.16	0.23 ± 0.14	0.41 ± 0.22
Hypermetropia	0.04 ± 0.02	0.26 ± 0.12	0.21 ± 0.12	0.99 ± 0.70

*RMS of posterior corneal aberrations (mean ± SD)*				
Low and moderate myopia	0.01 ± 0.02	0.06 ± 0.02	0.03 ± 0.01	0.06 ± 0.03
High myopia	0.04 ± 0.03	0.04 ± 0.03	0.06 ± 0.03	0.06 ± 0.05
Myopic astigmatism	0.01 ± 0.01	0.07 ± 0.02	0.06 ± 0.022	0.09 ± 0.04
Hypermetropic astigmatism	0.05 ± 0.07	0.07 ± 0.04	0.03 ± 0.05	0.08 ± 0.06
Hypermetropia	0.03 ± 0.02	0.02 ± 0.05	0.05 ± 0.01	0.05 ± 0.07

## Data Availability

The Excel sheet data used to support the findings of this study are available from the corresponding author upon request.
